# The New Patterns study: coordinated measures to combat child poverty

**DOI:** 10.1177/1403494820956452

**Published:** 2020-09-15

**Authors:** Eirin Mølland, Kristine L. Vigsnes, Tormod Bøe, Hilde Danielsen, Kjetil Grimastad Lundberg, Kristin Haraldstad, Torunn Alise Ask, Philip Wilson, Eirik Abildsnes

**Affiliations:** 1 School of Business and Law, University of Agder, Norway; 2 NORCE, Norwegian Research Centre As, Norway; 3 Kristiansand Municipality, Norway; 4 Department of Psychosocial Science, University of Bergen, Norway; 5 Department of Welfare and Inclusion, Western Norway University of Applied Sciences, Norway; 6 Department of Health and Nursing Science, University of Agder, Norway; 7 Department of Sociology and Social Work, University of Agder, Norway; 8 Centre for Rural Health, University of Aberdeen, UK

**Keywords:** Family interventions, social inequality, childhood poverty, low-income population, service coordination

## Abstract

**Background:**

Child poverty rates are rising in Norway with potential negative consequences for children. Services for families with low income are often fragmented and poorly integrated, and few coordinated initiatives have been implemented and evaluated in Norway.

**Aims:**

The aim of the current study is to evaluate how integrated and coordinated services provided over a prolonged period by a family coordinator are related to changes across a wide range of health, wellbeing and home environment indicators for the participants.

**Methods:**

The study uses a mixed methods approach utilising survey and register data, as well as information from interviews and shadowing, to document and evaluate outcomes associated with the intervention and the process of implementation. Data are gathered at baseline and annually throughout the duration of the study. Participants are identified to facilitate longer-term follow-up using register data.

**Conclusions:**

This project will develop important knowledge about the implementation of coordinated services to families with a low income, and how this way of organizing services influences important outcomes for the family members in the short and long term.

## Background

Child poverty rates are rising in Norway. The proportion of children living in low-income households has increased from 7.7% (2008–2010) to 10.7% (2015–2017) [1]. Growing up poor is associated with poorer physical and mental health, more developmental delay and lower school achievement [2, 3]. These adverse associations with low income have also been documented in studies of Norwegian youth [4–6], even though absolute deprivation to the extent of lacking basic amenities such as food and housing is uncommon [7].

Growing up poor influences children mainly indirectly, through pernicious influences on family processes, restricting opportunities for participation and through accumulated exposure to circumstances that may jeopardise healthy development [8–10]. Childhood poverty is associated with distal negative consequences, independent of adult socioeconomic status and financial wellbeing [11, 12], and may carry across generations [13, 14]. Intergenerational transfer has also been observed for income and education level, use of social support schemes [15–17], and it appears that those in the lowest ranks of parental earning are most negatively affected [14].

Poverty influences health and development through factors operating at several levels (i.e. individual, relational and institutional) [18], but services to children and families with low income are often poorly integrated and coordinated. The Cross-Departmental Review of Services for Young Children recommended that community-based programmes to combat poverty should: (a) involve parents as well as children; (b) be non-stigmatising; (c) be multifaceted; (d) last long enough to make a real difference; (e) be locally driven and involve parents and local communities; and (f) be culturally appropriate and sensitive to the needs of the parents and children [19]. Few such coordinated initiatives exist in Norway, and even fewer have been thoroughly evaluated [20]. In contrast, studies from Norway suggest that interventions often target individual problems or only individual members of the family, with users expressing frustration and powerlessness that no one addresses the ‘whole picture’ of their situation [21]. In the European context approaches addressing both children and parents are identified as the most effective at addressing social inequalities in children’s health and development [22]. In addition, the involvement of parents and developing a long-term relationship of trust between service providers and the families are identified as key elements for success [23].

A large randomised trial has been conducted in Norway studying the effect of comprehensive follow-up for low-income families [24]. The intervention aimed to increase parents’ participation in the labour market, the financial and housing situation of the family as well as the social inclusion of children [25]. The results indicate no significant effect of the method compared to standard follow-up. The majority (78%) of families participating in the randomised controlled trial (RCT) were immigrant families, therefore the results may not transfer well to non-immigrant families with low incomes.

Building on the existing literature and in an attempt to provide better services to low-income families, the innovative developmental project New Patterns has been developed. New Patterns recruits families with low income and a need for long-standing welfare services. Included families receive integrated welfare services through a family coordinator (FC) who coordinates services across sectors (culture, education, welfare, health and social services) and volunteer organisations, and supports all family members for 5 years. FCs tailor services to needs identified by the end-users through developing a family plan in which the question ‘what is important for you?’ is key, and by keeping the end-users’ perspectives as a premise when developing measures and aims for achieving a better situation for the families. New Patterns pays particular attention to children and youth across different arenas, such as childcare, early childhood education, school, leisure activities and the home.

### Objective

This paper describes the protocol for a repeated measures study that examines the outcomes associated with the provision of integrated and coordinated services for an extended time period by a FC to low-income families. The effectiveness of this intervention will be assessed with regard to several indicators of socioeconomic status and living conditions, service use, mental health and health-related quality of life, self-efficacy, school performance and leisure time activity participation (for details, see Table I).

**Table I.** Overview of instruments and data collection procedures in the New Patterns project.

	Data source	Subject	Type of information	Instrument or method	Time point						
					Enrolment	1 year	2 years	3 years	4 years	5 years	10–15 years
Quantitative	Children/ parents	Children age 0–17	Background^a^	Questionnaire (baseline)	x						
	Children/ parents	Children age 0–17	Social circumstances^b^	Questionnaire (yearly)	x	x	x	x	x	x	
	Parents	Children age 4–11	Child behaviour	SDQ^30, 46^	x	x	x	x	x	x	
	Children	Children age 11–17	Child behaviour	SDQ	x	x	x	x	x	x	
	Children	Children age 8–17	Quality of life	KIDSCREEN^31, 32^							
	School records	Children	School results	Register data	x	x	x	x	x	x	x
	Register data	Children	Socio economic status^c^	Register data							x
	Adults in the family	Adults age 18+	Background^a^	Questionnaire (baseline)	x						
	Adults in the family	Adults age 18+	Social circumstances^d^	Questionnaire (yearly)	x	x	x	x	x	x	
	Adults in the family	Adults age 18+	Quality of life	EQ-5D^28^	x	x	x	x	x	x	
	Adults in the family	Adults age 18+	Self-efficacy	GPSES^29^	x	x	x	x	x	x	
	Register data	Adults	Register data^c^	Register data							x
Qualitative	Family coordinator	Family coordinator (*N*)		Shadowing		x	x				
	Municipality contact	Municipality contact		Focus group interview		x	x				
	Families	Parents (*N*)		Interview		x	x				

SDQ: strengths and difficulties questionnaire; GPSES: generalised self-efficacy scale.

a Gender, year of birth, immigration information, role in family.

b Participation in kindergarten, after school programme and leisure activities (including what type of activity and regularity), relocation history, use of services, living arrangement.

c School results, labour market history, income.

d Marital status, size of household, relocation history, income, debt, work status, daytime activity, use of services, contact with voluntary services, education (including ongoing), standard of housing.

## Methods

Our study design is informed by the Medical Research Council’s recommendations for designing and evaluating complex interventions [26]. We shall apply a mixed-methods design with both quantitative and qualitative research methods, including questionnaires, register data, individual and focus group interviews and shadowing.

### Setting

The intervention was developed in Kristiansand municipality and funded through ordinary budgets and extraordinary funding from the Directorate of Labour and Welfare, and the ‘Public health programme’ initiated by the Norwegian Directorate of Health. The first FCs started working in Kristiansand in October 2015 and are employed by the Norwegian Labour and Welfare Administration (NAV). NAV governs welfare and social security benefits (including pensions) and active labour market policies in Norway and administers approximately one-third of the Norwegian national budget in allowances and services. By January 2020, 12 municipalities in southern Norway had elected to participate in the study. Families, FCs and important municipal stakeholders from four of the participating municipalities were invited to take part in focus groups, individual interviews and shadowing (Autumn 2019) and will be invited again (Autumn 2020). In addition, the researchers have taken part in workshops arranged for New Patterns FCs and will take part in future workshops.

### Participants

**Figure 1.**
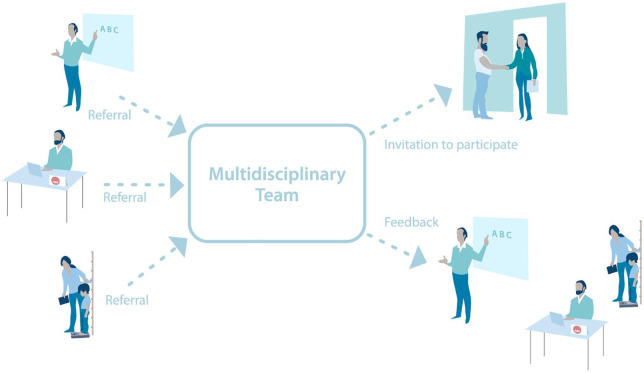
Schematic overview of the recruitment procedure in New Patterns. Families can be referred anonymously for discussions in the multidisciplinary team from multiple sectors such as school, the Norwegian Labour and Welfare Administration (NAV) or public health clinics. Following the discussion in the multidisciplinary team, families may be invited to participate in New Patterns and are then identified. In cases when families are not recruited to the project, the team provides feedback and advice for further action to the sector who sent the referral. In this case, families remain unidentified.

New Patterns recruits families with children aged 0–17 years, with household income averaged over 3 years below 60% of the equivalent median income in the population. In 2017, this was approximately €47,000 [27] for a family consisting of two adults and two children. In addition, family members must be in need of long-standing welfare services. Families are referred to New Patterns from different service sectors within the municipality; that is, kindergarten, school, public health clinics, general practitioners, NAV, child protection services and mental health services. Every referral to the project is discussed anonymously in a multidisciplinary team (see Figure 1), consisting of members representing different services in the municipality; that is, NAV, child protection services, mental health services and FCs. In the smallest municipalities, anonymous discussions are not feasible, and the discussions are based on consent from parents. If the multidisciplinary team concludes that the families could benefit from coordinated services provided by New Patterns, and there is capacity to include them in the project, the family is invited to participate.

When inviting new families to the project, the team attempts to include a purposive sample representing the diversity of the target population. Hence, we aim to include families that are different in terms of immigration background, size and family type. Families are not eligible for the project if the child protection service is considering taking over the daily care of the child/children at the time of recruitment. However, involvement of the child protection service as a support for the family is not an exclusion criterion. Families that move out of the municipality or which no longer include children younger than 17 years of age will leave the project and no longer receive follow-up by the FC. When needed, FCs will use translator services.

If the multidisciplinary team concludes that the family’s needs can be handled in ordinary services or there is not capacity in the project to include more families, the team provides feedback and advice for further action to the sector which sent the referral. We do not gather further information about the families that were not included in New Patterns. This is due to the anonymous referral process in which the identity of cases is only revealed when/if accepted into the intervention. We expect 200 families to be enrolled by the end of 2020 as the interventions are being scaled up in the participating municipalities. In the included municipalities, we expect to include 5.5% of families living with persistent low income in New Patterns, although it should be noted that many families with low income would not be eligible for the New Patterns intervention.

### Intervention

**Figure 2.**
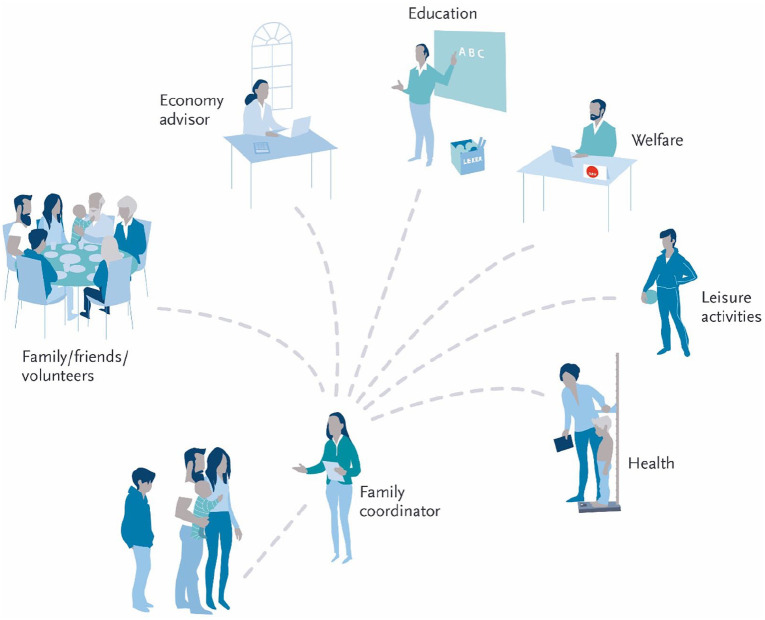
Schematic overview of how services to recruited families are integrated by the family coordinator. The family coordinator provides integrated services from different sectors; that is, culture, education, labour and welfare services, health and social services and volunteer organisations to the families included in the project.

The intervention includes a close follow-up of both adults and children in participating families over a period of 5 years. Included families receive integrated welfare services from a permanent FC who coordinates services from different sectors; that is, culture, education, labour and welfare services, health and social services and volunteer organisations (see Figure 2).

One FC is responsible for following up 10 families for 5 years. When new families are included, the FC performs a detailed survey of the family members’ different needs, enabling targeted and appropriate help to the family. The intervention is tailored to what each family experiences as their needs. The FC works with the family on the domains of the family’s everyday life, offering home visits and accompanying the family members to meetings with, for example, general practitioners, NAV, school, kindergarten and voluntary organisations.

The first FCs have been important in developing the project, in particular developing the systematic mapping. This mapping includes information about income, education, living conditions, leisure activity, work, health and wellbeing. Mapping is performed when new families enter the project and is then repeated in subsequent years. An important aim has been to make this mapping useful for the families, FCs, the intervention itself and for research purposes. It provides information needed to develop the ‘family plan’, a coordination tool used by the FC in their work. The family plan is based on all family member’s needs, challenges, resources and the question ‘What is important to you and your family?’ Based on the preliminary experiences from the pilot, the family plan often contains topics such as housing, economy and leisure activity. In most families, employment is a long-term goal for the adults.

The FCs play a key role in the intervention. Besides formal education within social work or child welfare services, the FCs must be able to exercise leadership and coordinate complex inter-sectoral work as well as possessing a good overview of the different systems they coordinate on behalf of the families. All FCs are invited to take part in a professional network including mentoring, sharing competence and experiences. As elsewhere in Norway the municipalities included in the intervention represent diversity with respect to size, centralisation and the availability of different services.

### Quantitative methods

The detailed mapping described as part of the intervention makes it possible to provide quantitative insight on children and families in poverty. We shall assess all participants in the intervention at enrolment and annually for the duration of the project using surveys for parents and children.

Table I provides an overview of the data collection in the study, including both quantitative and qualitative measures. In parent surveys we collect information about education level, work experience, employment status, immigration status, household income, housing and other relevant expenses, debts, relocations and the suitability of housing and the use of healthcare and social services. We have included Norwegian translations of validated instruments to measure health-related quality of life (i.e. EQ-5D-5L [28]), and self-efficacy (i.e. the general self-efficacy scale (GSE) [29]). The surveys relating to children assess childcare attendance, after-school activities, leisure time activities, skills in reading and mathematics as well as results when finishing primary education, completion of upper secondary education, the use of healthcare services and, when relevant, contact with child welfare services. We have included validated instruments to assess mental health (i.e. the strengths and difficulties questionnaire (SDQ) [30]) and health-related quality of life (i.e. KIDSCREEN-27 [31, 32]).

We have measured the same outcome variables over time, providing us with a unique panel dataset consisting of information about the participants before and during inclusion in New Patterns. Exploiting the panel structure of the data set, we will be able to estimate the effects of New Patterns, investigating whether the coordinated services are associated with improved health, welfare and quality of life of the participants. In principle, the estimation strategy will be a comparison of status before and after the interventions for the participants, controlling for individual fixed effects [33]. Furthermore, we will capitalise on the longitudinal data from the yearly surveys to investigate stability and the change in outcome measures as the intervention proceeds using statistical tools such as paired-samples tests and general linear mixed modelling approaches, accounting for independence violations as well as the clustered structure of data. Missing data will be handled using multiple imputation and modelling methods that utilise full information maximum likelihood estimation. For psychometric analyses of instruments, we will use structural equation modelling approaches (i.e. confirmatory factor analyses).

Baseline data will provide important descriptive characteristics of the participating family members, which can be compared to national and international population norms, as well as national register data. The intervention is complex and as we study the effect on multiple outcomes we will adjust for multiple hypothesis testing [34, 35]. The study is designed for estimating long-term (5–10 years after New Patterns) effects on education and labour market outcomes using Norwegian register data, we will be able to identify offspring who participated in New Patterns as well as potential control groups. The register data will provide information about individual background characteristics (i.e. if they grew up with persistent low income and parents were unemployed, as well as demographic information and municipality of residence) and important outcome variables (such as education and labour market engagement). In order to identify long-term causal effects, we will capitalise on the staggered roll-out of the intervention and the fact that there are municipalities in the region that have not implemented the intervention. Hence, difference-in-difference approaches are feasible [33, 36]. This method relies on the assumption of a common trend in the outcome variables, if this assumption fails, propensity score matching prior to the difference-in-difference approach will be performed [37]. In addition, matching on observable characteristics, such as socioeconomic background, will also be feasible [38].

Nationally and internationally, few service coordination interventions have been evaluated with regard to their effects [20]. An evaluation report from the UK-based Sure Start programme provided evidence of positive changes; for example, less harsh discipline, more stimulating and less chaotic home environments, with small to medium effect sizes [39] ranging from 0.17 to 0.66 [40]. Based on these data, we calculated sample size requirements for detecting similar effect sizes for dependent means (matched pairs) given levels of *α* with a statistical power of 80%. These estimates suggest we can detect medium effect sizes with 34 participants and small effect sizes with 199 participants at a *α* level of 0.05. A more stringent *α* criterion of 0.01 increases the sample size requirement to 50 and 296 for medium and small effect sizes, respectively. However, it is important to acknowledge that the power calculations are only estimations and depend on the assumptions made in the calculations. Based on the plans for enrolment, at least 200 families will be participating in the New Patterns. Family characteristics for those already enrolled suggest that the mean number of children per family is 2.6 (mode 2), and the mean number of adults is 1.4 (mode 1) suggesting an estimated range of 400–600 children and 200–300 adults in the project at large. The sample has an approximate 50/50 split with regard to ethnicity, which will allow us to compare and contrast participants with Norwegian and non-Norwegian origins.

### Qualitative methods

In the qualitative study we shall compare different FC roles and practice patterns within different institutional settings. New Patterns is not only focused on making change in the situation of the participating families. The programme also aims to promote welfare innovation through increasing coordination and knowledge sharing across services, cooperation with the civil sector and taking into account the families’ own perspectives of what they need. The qualitative research will assess how this ambition is realised in practice in New Patterns.

The qualitative part of the study will explore the impact of the integrative and holistic approach in New Patterns, and how end-users in low-income families experience being part of this project. The study investigates how services and organisations can ensure that they maintain a child perspective and a caretaker perspective as well as how the strengths and perspectives of the families can be utilised and developed in order to improve their situation. A strategic sample of service providers, civil sector organisations and end-users from rural as well as urban municipalities will be interviewed individually or in focus groups. This will secure a wide range of experiences. In order to map the municipal coordination strategies, we will spend time at the locations where the FCs work, and interview as well as follow the central actors in their work, using the method of shadowing [41]. In this way we will study how the holistic perspective is maintained and potential dilemmas solved when providing coordinated services from different sectors to parents as well as children.

### Mixed methods

Qualitative and quantitative data are collected in a parallel mixed methods design [42]. Together with the participating municipalities, we will conduct workshops throughout the study. Users’ and professionals’ experiences are essential to ensure the feasibility of New Patterns. This bottom-up input in combination with data generated from qualitative and quantitative methods will facilitate the implementation of New Patterns. Based on the qualitative research and input from participants in the workshops we will study how New Patterns and the role of the FC and the detailed mapping can be adopted in ordinary services in different contexts. Moreover, insight generated through the innovation will be communicated to the management of welfare services as well as politicians and provide potential for further innovation and development of welfare services.

### Ethics

Participation in New Patterns is voluntary, and the services provided to the families are not contingent on participation in the research project. The study is conducted according to recommendations from the Norwegian Data Protection Services (file numbers 282648 and 27435). The confidentiality and anonymity of the participants will be protected during data management and in publications and dissemination from the project.

## Results

The current sample consists of 54% with an immigrant versus a Norwegian background, 59% single versus two-parent families, and the number of children in the families ranges from one to eight. By including a purposive sample, the feasibility of the model will be tested in families with different compositions, backgrounds and challenges. All invited families, in addition to a low income, have a complex life situation and long-standing need for coordinated services so the project targets a particularly vulnerable group. To illustrate this, in the current sample 90% of the families live in rented accommodation and 78% do not participate in the labour force, while corresponding national statistics for all families living with consistent low income indicate that 62.1% live in a rented home and 58.8 % do not participate in the labour force [27].

## Discussion

We have limited knowledge about life circumstances for families with children growing up with a persistent low income in Norway. This study will provide new knowledge about a population segment that is underrepresented in surveys. The innovation is tested out in rural as well as urban municipalities of different sizes and organisation, and the participating families have diverse challenges. Hence, the feasibility of New Patterns will be relevant beyond the participating municipalities. Currently, services are fragmented and uncoordinated [43]. New Patterns does not introduce new services but coordinates the optimal use of existing services. This may have the potential to maximise the benefit of welfare services to families and individuals who have a complex life situation. Through taking into account families’ own perspectives and needs when prioritising and delivering services it is expected that these services may become more relevant, precise and therefore also more effective. Utilising existing services increases the potential for implementing innovation in ordinary services in different contexts beyond the project period. We regard the detailed mapping as part of the intervention, as family members as well as FCs will have access to more comprehensive information than would normally be available to service providers. Hence, services can be tailored accordingly. The intervention might be particularly effective for non-Norwegian-speaking families and other families who are unlikely to understand the way services operate, and who will have particular difficulty navigating their way through services.

Although we recognise that a RCT is the gold standard for identifying causal effects [33], this design is not always feasible or the best option [44, 45] for studying the effect of interventions being implemented in a complex real-world setting. In our study the participants are not randomly selected. In order to represent the diversity in the target population we instead aimed at including a purposive sample with respect to family structure and background. A threat is the possibility of recruiting families in which the potential for success is high while avoiding the recruitment of families with more complex needs. To counteract ‘cherry-picking’ and addressing this threat, every referral is discussed in a multidisciplinary team whose mandate is to secure the diversity of included families. Preliminary analyses also confirm that all invited families have a complex life situation and long-standing need for coordinated services.

It is challenging to identify potential control groups that provide information about the counterfactual outcome of the families. In this study we have information about the participants at baseline as well as during and after treatment. In addition, when assessing the long-term effects using Norwegian administrative register data, we will be able to compare with potential control groups as discussed in the method section. The mixed-method design will allow the integration of qualitative and quantitative data that can provide extended knowledge beyond what separate analyses would provide.

## Potential of the study

In this project we will develop important knowledge about the implementation of coordinated services to families with a low income, and how this way of organising services influences important outcomes for the family members in the short and long term. We aim to contribute to better collaboration among services in different sectors, to improve access, quality and utility of services to families with low incomes through research-based knowledge. The mapping of end-users’ experiences will contribute to understanding both the barriers to achieving a better situation and what means they see as helpful. This process will help with targeting these families in more effective ways. Furthermore, we aim to increase participation in work and society for families with low incomes, thereby contributing to reduced social inequality in health and welfare.
